# Further Support to the Uncoupling-to-Survive Theory: The Genetic Variation of Human *UCP* Genes Is Associated with Longevity

**DOI:** 10.1371/journal.pone.0029650

**Published:** 2011-12-27

**Authors:** Giuseppina Rose, Paolina Crocco, Francesco De Rango, Alberto Montesanto, Giuseppe Passarino

**Affiliations:** Department of Cell Biology, University of Calabria, Rende, Italy; University of Valencia, Spain

## Abstract

In humans Uncoupling Proteins (UCPs) are a group of five mitochondrial inner membrane transporters with variable tissue expression, which seem to function as regulators of energy homeostasis and antioxidants. In particular, these proteins uncouple respiration from ATP production, allowing stored energy to be released as heat. Data from experimental models have previously suggested that UCPs may play an important role on aging rate and lifespan. We analyzed the genetic variability of human *UCP*s in cohorts of subjects ranging between 64 and 105 years of age (for a total of 598 subjects), to determine whether specific *UCP* variability affects human longevity. Indeed, we found that the genetic variability of *UCP2, UCP3* and *UCP4* do affect the individual's chances of surviving up to a very old age. This confirms the importance of energy storage, energy use and modulation of ROS production in the aging process. In addition, given the different localization of these UCPs (UCP2 is expressed in various tissues including brain, hearth and adipose tissue, while UCP3 is expressed in muscles and Brown Adipose Tissue and UCP4 is expressed in neuronal cells), our results may suggest that the uncoupling process plays an important role in modulating aging especially in muscular and nervous tissues, which are indeed very responsive to metabolic alterations and are very important in estimating health status and survival in the elderly.

## Introduction

Un-Coupling Proteins (UCPs) belong to a family of anion transporters located in the inner membrane of mitochondria responsible for uncoupling substrate oxidation from ATP synthesis. As a consequence stored energy is released as heat. To date, five UCP homologues, *UCP1* to *UCP5*, have been identified in mammals. *UCP1* (4q28.31), the first member characterized, is predominantly expressed in brown adipose tissue (BAT), where it has a well-established role in cold- and diet-induced thermogenesis [1]. *UCP2* and *UCP3* share a common region on chromosome 11q13. *UCP2* is widely expressed in many organs and tissues [2], while *UCP3* is principally expressed in skeletal muscle, cardiac muscle, and BAT [3], [4]. Finally, *UCP4* (6p12.3) and *UCP5* (Xp24) are predominantly expressed in the central nervous system and at a lower level in other tissues [5], [6]. Unlike UCP1, the physiological role of the other UCPs still remains to be fully elucidated. It has been hypothesized that these proteins may provide protection from oxidative damage by preventing excessive production of mitochondrial Reactive Oxygen Species (ROS) [7], [8]. Indeed, Ucp-knockout mice have increased levels of ROS and show signs of increased oxidative damage [9]–[11]. Further evidence indicates that activation of *UCP2* and *UCP3* by ROS leads to a mild uncoupling and to diminished ROS formation [12], [13], while their inhibition by purine nucleotides increases membrane potential and mitochondrial ROS production [14].

The uncoupling activity of UCPs has also been linked to regulation of other more specific metabolic functions [15], [16] such as fatty acid oxidation [17], glucose-stimulated insulin secretion [18], whole body energy balance [19], and apoptosis [20]. Human aging is characterized by a gradual reduction in the ability to coordinate cellular energy expenditure and storage (crucial to maintain energy homeostasis), and by a gradual decrease in the in the ability to mount a successful stress response [21], [22]. These physiological changes are typically associated with changes in body composition (i.e increase in fat mass and the decline in fat-free mass), and with a chronic state of oxidative stress with important consequences on health status [23]–[25]. Mitochondrial function is crucial in these processes, being mitochondria the main cellular sites controlling energy metabolism and the redox state. Thus, by promoting fatty acid oxidation, and by reducing ATP and ROS production, the induction of mitochondrial uncoupling through UCPs may also be a critical pathway in the modulation of the rate of aging and lifespan [26]–[28]. This idea, first proposed by M. D. Brand [26] and termed “uncoupling-to-survival”, is now supported by several experimental evidences (for reviews see: [27], [29], [30]). Of interest, targeted expression of exogenous UCP extends lifespan of adult flies [31], [32], while mice with higher metabolism live longer and have higher uncoupling activity [33]. Transgenic and knockout mice for *Ucp* genes show alterations in lifespan [34], [35]. Interestingly, it has also been shown that *in vivo* uncoupling mimics metabolic and lifespan effects of calorie restriction (CR) [36]. Consistently, mice subjected to CR show an increased expression of *Ucp2* and *Ucp3* [37], [38].

In humans the correlation between mitochondrial uncoupling and aging emerges from association studies which support a role for UCPs in many age-related phenotypic traits involving alterations in cellular energy homeostasis such as obesity, diabetes and lipid-related diseases [39]–[41]. We recently performed a population-based study in a human cohort of elderly subjects which demonstrated that *UCP3* variability has an impact on hand grip strength, one of the most important hallmarks of human aging [42] and more recently we also found that two variants (A-3826G and C-3740A) in the upstream enhancer region of human *UCP1* gene affect the expression of the gene and are correlated with human longevity [43].

Given the tissue-specific expression of *UCP* genes, it seems likely that they may affect the senescence of different tissues with important effects on the overall aging process and consequently on lifespan. Thus, the goal of the present study was to assess both the primary effects of single loci and gene-gene interactions to test the hypothesis that other *UCP* genes may contribute to survival at very old age either independently and/or through complex interactions.

## Methods

### Ethical statement

Samples were collected within the framework of several recruitment campaigns carried out for monitoring the quality of aging in Calabria from 2002.The recruitment campaigns and subsequent analyses received the approval of the Ethical committee of the University of Calabria. All subjects provided written informed consent for studies on aging carried out by our research group. White blood cells (WBC) from blood buffy coats were used as a source of DNA.

### The Sample

A total of 598 subjects (293 men and 305 women, age range 64–105 years; mean ages 82.74 (±11.66) and 85.23 (±10.84) years, respectively) participated in this study. All subjects were born in Calabria (southern Italy) and their ancestry in the region was ascertained up to the grandparents' generation. Younger subjects were contacted through family physicians. Subjects older than 90 years were identified through the population registers and then were contacted by specialized personnel and invited to join the study. Each subject underwent a medical visit carried out by a geriatrician who also conducted an interview including the administration of a structured questionnaire, validated at European level. The questionnaire collected socio-demographic information, evaluated physical and cognitive status, and self-reported health status. Subjects with dementia and/or neurologic disorders were not included.

The analyses were carried out by dividing the sample into two specific age classes obtained according to the survival function of the Italian population from 1890 onward [44]. The two “thresholds of longevity” used to define these age classes were 88 years for men and 91 years for women.

### SNPs selection

Polymorphisms within the *UCP* genes were selected from literature data, and using information from online databases such as NCBI dbSNP and HapMap. SNP selection was based on allele frequency, position, and functional effects. For each gene the selected SNPs are reported in Table 1.

**Table 1:** Description and localization of selected SNPs in the UCP genes.

Gene symbol	dbSNP ID		Physical location	Function annotation *
UCP2	rs660339	C/T	exon 4	Ala55Val
	rs659366	G/A	5′-proximal region	−866 G/A
UCP3	rs15763	C/T	3′UTR	
	rs1800849	C/T	5′-proximal region	−55 C/T
UCP4	rs9472817	C/G	intron 8	
	rs10498769	C/G	5′UTR	
UCP5	rs2235800	A/T	intron 7	
	rs5975178	C/T	5′-proximal region	

*Provided only for coding SNP (amino acid residues for two alleles) and for SNPs in the 5′ flanking region (the nucleotide positions relative to the transcription start sites in the promoter regions are indicated).

### Genotyping

DNA was prepared from peripheral blood lymphocytes using standard techniques. Genotyping of the eleven polymorphic sites was carried out using a TaqMan Real Time PCR (SNP Genotyping kit, Applied Biosystems). In all assays the fluorescent FAM dye was used to label the wild-type allele, while the mutant allele was labeled by fluorescent VIC dye. PCR reactions were carried out in a total volume of 5 µl containing 20 ng of genomic DNA, 2.5 µl of TaqMan Universal Master mix (concentration of 2×), 0.25 µl of Custom TaqMan SNP Genotyping Assay (concentration of 20×) containing both primers and probes. The amplification protocol (60°C for 30 seconds, 95°C for 10 minutes followed by 40 cycles at 95°C for 15 seconds and 60°C for 1 minute) was performed by using a StepOne thermal cycler (Applied Biosystems). Random regenotyping was conducted to confirm the results.

### Genetic and statistical analyses

For each polymorphism of the *UCP* genes, allele frequencies were estimated by counting genes from the observed genotypes. The Hardy-Weinberg equilibrium (HWE) was tested using the exact test proposed by [45]. Standard errors for alleles were computed according to the hypothesis of the multinomial distribution.

### Single-locus analysis

In recent years different robust tests have been proposed to test genotype-phenotype associations in case-control studies. One of these is the MAX3 test which assumes the maximum of three different Cochran-Armitage trend tests (CATTs) evaluated with respect to three different genetic models of inheritance: dominant, additive or recessive [46], [47]. The main drawback of such tests is represented by the fact that they could be seriously affected by confounding factors [48]. In this context, So and Sham recently proposed a robust association test allowing for quantitative or binary traits as well as covariates [49]. This test was based on the score test and has been implemented in the R package *RobustSNP*. In the present study the robust association test proposed by So and Sham was applied to estimate the association between the variability in the *UCP* genes and the probability of reaching advanced ages. In each test we included sex and Body Mass Index (BMI) as covariates.

### Haplotypic analysis

Pairwise measures of linkage disequilibrium (LD) between the analyzed loci were calculated with the Haploview 4.2 [50]. The amount of LD was quantified by Lewontin's coefficient (D').

In order to model the effect of the *UCP* haplotypes on the probability to attain longevity we used the *haplo.stats* package of R. It implements a haplotype-based association analysis within the generalized linear model (GLM) framework that allows for ambiguous haplotypes. The *haplo.score* function of this package has been used to obtain the GLM-based score statistics for testing global and individual haplotype effects on the probability to attain longevity [51]. Permutation-based p-values were used to evaluate the significance of the scores obtained (10000 permutations). In this model the effect of the different haplotypes was assessed assuming a dominant model after adjusting for sex and BMI.

### Interaction analysis

In order to explore the interaction effects between the analyzed polymorphisms on the probability to be part of the very old age group, the Model-Based Multifactor Dimensionality Reduction (MB-MDR) method was applied [52]. In this model only all possible second order interactions, after adjusting for main effects sex and BMI, were analyzed.

All statistical analyses were performed using *genetics*, *RobustSNP*, *mbmdr* and *haplo.stats* packages of R 2.10.1.

## Results

Table 2 reports the socio-demographic characteristics of the sample analyzed according to age group. The observed genotype frequencies (Table S1) were in agreement with those expected at HWE (p>0.01). Table 3 reports the results of the association test for the three different genetic models (dominant, additive and recessive) obtained in the analyzed sample using the RobustSNP algorithm. Table 3 clearly shows that the variability of the UCP polymorphism was significantly associated with the analyzed phenotype. In particular, using the minor allele for each SNP as reference and after adjusting for BMI and sex, the dominant model for the rs660339 (*UCP2*) and the rs1800849 (*UCP3*) resulted to be significantly associated with the longevity phenotype (p = 0.001 in both cases), while the recessive model was the most likely for SNPs rs15763 (*UCP3*), rs9472817 (*UCP4*) and rs2235800 (*UCP5*) (p<0.05). However, after adjusting for multiple testing, all the previous associations remained statistically significant, except those for rs2235800 of the *UCP5* gene (p = 0.058).

**Table 2:** Socio-demographic characteristics of the analysed sample according to sex and age group.

Younger group	Males	Females	Total
*N (%)*	155 (41.3)	220 (58.7)	375 (100)
*Mean age (SD)*	72.86 (6.027)	80.64 (9.127)	77.42 (8.858)
*BMI (SD)*	27.06 (4.122)	26.38 (4.845)	26.66 (4.566)
Older group	Males	Females	Total
*N (%)*	138 (61.9)	85 (38.1)	223 (100)
*Mean age (SD)*	93.85 (3.771)	97.09 (3.235)	95.09 (3.903)
*BMI (SD)*	24.42 (3.701)	22.50 (3.994)	23.69 (3.918)

Note: age-cut-offs to define the younger and older groups were 88 years for males and 91 years for females, as reported in Materials and Methods section.

SD: standard deviation.

**Table 3:** Results of the Robust SNP association test for the most likely genetic model (additive, recessive and dominant) obtained in the analyzed sample.

Gene	SNP	MAF	Z(ADD)	Z(REC)	Z(DOM)	P(ADD)	P(REC)	P(DOM)	P-value*
UCP2	rs659366	A (0.295)	0.936	0.338	0.978	0.349	0.735	0.328	0.555
	rs660339	T (0.341)	3.049	1.477	3.190	0.002	0.140	0.001	0.003
UCP3	rs15763	T (0.247)	2.163	2.468	1.454	0.031	0.014	0.146	0.029
	rs1800849	T (0.099)	2.788	−1.197	3.186	0.005	0.231	0.001	0.003
UCP4	rs9472817	G (0.498)	−1.984	−3.607	0.316	0.047	<0.001	0.752	0.001
	rs10498769	G (0.190)	−0.085	−0.660	0.130	0.933	0.509	0.897	0.761
UCP5	rs2235800	A(0.387)	1.554	2.120	0.849	0.120	0.034	0.396	0.058
	rs5975178	C (0.474)	0.203	0.210	0.170	0.839	0.833	0.865	0.959

Z(ADD), Z(REC) and Z(DOM) are the z-statistics under the additive, recessive and dominant models respectively. P(ADD), P(REC) and P(DOM) are the p-values under the three genetic models.

*p-value was adjusted for multiple testing of different genetic models obtained by the proposed analytic approach.

MAF: Minor Allele Frequency.

From the z-statistics of these models we observed that carriers of the T allele at rs660339 and at rs1800849 (dominant models) significantly influenced the probability to be part of the oldest group (positive scores). For the recessive models, homozygous subjects for the T allele at rs15763 variation also increased the probability to be part of the oldest group, while this probability was significantly decreased (negative score) for homozygous subjects with the T allele at rs9472817.

By using the MB-MDR approach we did not find any significant interaction effect among the analyzed polymorphisms (data not shown).

**Figure 1:**
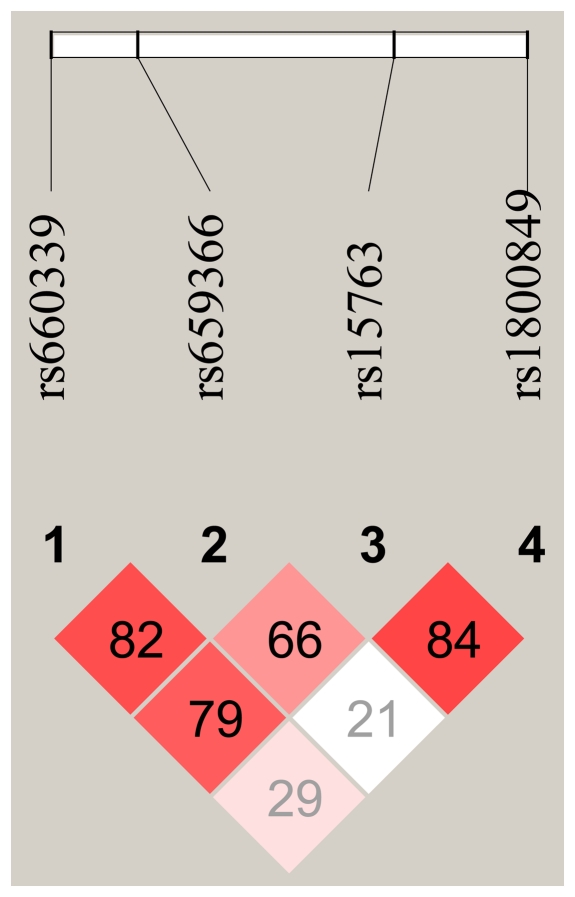
Schematic representation of linkage disequilibrium (D' coefficient) among the four SNPs of the UCP2-UCP3 gene cluster.

Subsequently, using the aforementioned GLM-based algorithm, we evaluated the effect of the *UCP* haplotypes on the probability to attain longevity. LD analysis showed that among the four SNPs in the *UCP2-UCP3* gene region rs660339, rs659366 and rs15763 were in moderate LD (see Fig. 1), while rs1800849 was in LD with rs15763 (D' = 0.83), but it was virtually unlinked with the others (D'<0.30). Finally, we found a weak LD between rs9472817 and rs10498769 in the *UCP4* gene (D' = 0.47), and between rs2235800 and rs5975178 in the *UCP5* gene (D' = 0.44). Based on these LD patterns, and using a D' cutoff equal to 0.6, haplotypes were reconstructed at the *UCP2*-*UCP3* and *UCP3* loci.

As shown in Table 4, the analysis of the *UCP2-UCP3* haplotypes revealed that the CAC haplotype, consisting of the minor allele for rs660339, major allele for rs659366, and minor allele rs15763, was associated with a decreased probability of attaining longevity (P =  0.003), while the TGT haplotype (opposite combination of alleles) acted in a reverse fashion by increasing this probability (p = 0.046). However, the strength of these associations was influenced by the allelic status at rs659366 of the relevant haplotype. Indeed, the CGC and TAT haplotypes, when compared to the above, differ only for rs659366 and did not show any effect. For the *UCP3* haplotypes involving rs15763 and rs1800849, we found that carriers of the CC haplotype have a decreased probability of achieving longevity (P<0.001). Conversely, as previously reported in the single-locus analysis, the presence of a T allele at either rs15763 or rs1800849 increased such probability, although this positive effect was more pronounced when the T allele at rs1800849 was involved (p = 0.044 *vs* P = 0.006).

**Table 4:** Estimation of haplotype frequencies in the *UCPs* SNPs and association with longevity in the analyzed sample.

Gene	SNPs			Freq^a^	Score	P-value^b^
UCP2-UCP3	rs660339	rs659366	rs15763			
	C	A	C	0.029	−2.876	0.003
	C	G	C	0.596	−1.747	0.086
	C	G	T	0.028	0.317	0.754
	C	A	T	0.004	0.877	0.400
	T	A	C	0.077	0.953	0.347
	T	G	C	0.050	0.971	0.339
	T	A	T	0.184	1.390	0.165
	T	G	T	0.030	1.985	0.046

UCP3	rs15763	rs1800849				
	C	C		0.659	−3.720	<0.001
	T	C		0.243	2.029	0.044
	C	T		0.095	2.766	0.006

a Estimated haplotype frequency.

b Monte-Carlo p-value from 10^4^ replications.

Note: p-values of global score statistics based on 10^4^ replications were 0.047 for UCP2-UCP3 haplotypes, 0.0028 for UCP3 haplotypes.

## Discussion

Substantial evidence suggests that the ability of UCPs to reduce ROS and regulate energy utilization underpins the ability of UCPs to promote lifespan in various experimental models [27], [28], [29], [30], [35]. In the present study we found that variants in *UCP2*, *UCP3*, and *UCP4* significantly affect an individual's chances of becoming ultra-nonagenarians. The different localization of the proteins we found associated with longevity allows us to predict the areas where the uncoupling process may play an important role in survival at very old age.

*UCP2* and *UCP3* genes cluster on chromosome 11q13 in a region linked to lower resting metabolic rate in humans [53], which is also syntenic to a region of mouse chromosome 7 linked to hyperinsulinemia and obesity [54]. Their chromosomal location and their association with fatty acid transport across mitochondrial inner membrane and β-oxidation, support the hypothesis that, in addition to functioning as regulators of oxidative stress, both UCP2 and UCP3 can modulate cellular energy metabolism [16], [55]. While mounting evidence has documented the importance of both *UCP2* and *UCP3* variability in the pathophysiology of different chronic metabolic diseases, such as diabetes and obesity [41], [56], relatively little is known of their implication in human aging and longevity. In this study we found that the *Ala*55*Val* (rs660339) of UCP2, and -55 C/T (rs1800849) and rs15763 of UCP3 were significantly associated with survival at very old age after Bonferroni correction.

*Ala*55v*al* SNP in exon 4 causes a conservative amino acid change that does not seem to cause a functional change in the protein. However, it has been reported that the *Val/Val* genotype causes a lower degree of uncoupling, an enhanced metabolic efficiency, and a lower fat oxidation than the *Ala*/*Ala* and *Ala*/*Val* genotypes [57]. In addition this genotype has been associated to higher exercise energy efficiency [58], higher risk in obesity, higher incidence of diabetes [59], [60], and a higher acute insulin response to glucose [61]. Moreover, individuals with the *Val/Val* genotype had greater weight loss and a higher BMI [62]. Nevertheless, other studies have produced conflicting results [63]–[66]. Here we show that this polymorphism also contributes to extend lifespan. Given the wide distribution of UCP2, it is likely that systemic effects on global energy metabolism and redox state underlie the association with longevity. However, *UCP2* also exhibits tissue-specific regulation suggesting tissue specific physiological effects. In this regard, evidence has been provided that in the brain, liver and other tissues UCP2 functions as regulator of oxidative stress [67]–[69], in the heart as a regulator of energy availability [70], in white adipose tissue and skeletal muscle as a regulator of fatty acid metabolism [71], [72], and as a regulator of insulin dynamics in pancreatic β and α-cells [73], [74]. In this scenario, variants of UCP2 may have positive and negative effects depending on the tissue and on the fine balance between energy production/consumption and mitochondrial ROS generation.

It is widely accepted that damage by free radicals to the molecules with which they come into contact are the underlying cause of aging, therefore the ability of UCP2 to attenuate steady-state levels of ROS strongly suggests an essential role in lifespan extension. Recently, Andrews proposed that UCP2 promotes longevity by shifting a cell towards fatty acid fuel utilization thus pointing to a major role of UCP2 in modulating metabolism [75]. This hypothesis is somewhat supported by Barbieri and colleagues [76] who analyzed the *Ala*55v*al* polymorphism in a human cohort of elderly subjects from an Italian population. Although the authors did not find an association with longevity, they found that individuals with the *Val/Val* genotype had significantly higher energy expenditure parameters. Interestingly, the A-IGF1R/*Asp*-IRS2/*Val*-UCP2 allele combination was associated with a better metabolic profile, higher energy expenditure parameters, and lower mortality rates in longevity, thus indicating a contribution of *Ala*55v*al* polymorphism to the regulation of energy balance and survival [76]. The latter and our finding suggest that the 55*Val* allele might promote longevity by conferring protection towards age-related decline of metabolic rate. It is indeed well-known that human aging is accompanied by a decrease in resting metabolic rate, the largest component of total energy expenditure, which significantly affects disability and morbidity among the elderly [24], [77]. On the other hand, long-lived subjects show a preserved metabolic profile being less prone to the metabolic deregulation normally occurring with aging [78]. Accordingly, Rizzo and colleagues [79] reported that compared to aged subjects, healthy long-lived subjects display energy expenditure parameters that are closer to the values of healthy middle-aged adults. In a recent study we demonstrated a correlation between the activity of UCP1 and human survival, and hypothesized a slower decline of energy expenditure with age at the basis of the correlation [43].

Skeletal muscle, where UCP3 is mainly expressed, contributes considerably to the basal metabolic rate [80]. We found that carriers of the TT genotype (rs15763), and carriers of the -55 T allele (rs180084) were significantly over-represented among long-lived subjects. The rs15763 is located in the 3′UTR of the gene and has no clear functional role so far; on the contrary, the -55 T allele has been associated with a significantly increased gene expression and has been shown to positively modulate the resting metabolic rate of skeletal muscle [81], [82]. In addition, the over-expression of UCP3 in muscle cells has been shown to be associated with decreased production of ROS as well as with facilitating fatty acid oxidation [17], [83]. The physiological status of the muscle mass reflects a complex equilibrium between nutrition, metabolism and the response to stress. Aging muscle is characterized by a progressive loss of mass and a gradual increase of weakness leading to sarcopenia, a condition associated with physical disability, and with an increased risk of developing disorders such as atherosclerosis, type II diabetes and hypertension [84]. Sarcopenia is closely linked to a decrease in resting metabolic rate as well as to mitochondrial dysfunction and oxidative stress [85]. Therefore, in the context of the proposed functions of UCP3, the physiological consequence of an increased UCP3 activity in skeletal muscle might be to slow down the age related decline of muscle performance as a result of decreased ROS production, an increased protection of mitochondria from lipid peroxidation, and better metabolic efficiency [28], [83]. Accordingly, it has been found that mild uncoupling has an impact on cellular aging in human muscles in vivo [86]. This is in keeping with our previous work, where we have shown that the hand grip strength, the most effective death predictor in the elderly, was higher in the carriers of rs1800849-T allele than in the remaining the population [42].

Haplotype analysis confirmed the single SNP analyses, and highlighted the importance of the entire *UCP2-UCP3* locus in human aging and longevity. In fact, one finding of interest emerging from this analysis is that the *UCP2*-rs659366 (-866 G/A), which was uninformative to single-locus analysis, seemed instead to be effective in modulating the aging process. In particular the rs659366G allele appeared to exert a modest beneficial effect on human survival. Indeed, the presence of the rs659366G allele reduced the negative effect of the rs659366C-rs15763C haplotype, while it increased the positive effect of the rs659366T-rs15763T haplotype. The rs659366 (-866G/A) variant changes promoter activity, affects metabolic rate and oxidative stress, and has been associated with metabolic traits in several, although not all, studies [87].

UCP4 and UCP5, together with UCP2, are known as neuronal UCPs due to their widespread distribution in the brain [6]. Neurons have a very high metabolic rate and consequently a high production of ROS. Andrews and co-workers observed that neuronal uncoupling activity leads to decreased ROS levels, a decreased Ca2+ voltage-dependent influx and increased local temperature in neuronal microenvironment [88]. We found that only the *UCP4* rs9472817-GG genotype negatively impacts on the probability to attain longevity. It has been shown that UCP4 activity can induce an adaptive shift in energy metabolism, from mitochondrial respiration to glycolysis, that helps sustain neurons under conditions of metabolic and oxidative stress [89], [90]. Therefore, by decreasing free radical production and stabilizing cellular calcium homeostasis, UCPs expressed in neurons may positively influence neuronal function (synaptic transmission and plasticity) and retard the cellular deterioration associated with aged-related neurological disorders. Accordingly, the expression of UCP4 has been found significantly reduced in brains of subjects affected by Alzheimer disease [91]. Moreover, it was found that CC genotype for rs10807344 of *UCP4* gene, not found to be associated in the present study with the longevity phenotype, exerts a protective effect on the occurrence of multiple sclerosis and of leukoaraiosis, a vascular demyelinization of the white matter of the brain [92], [93]. Thus, it is likely that variants affecting the UCP4 function might have consequences on the aging of the nervous system, and then might have a potential impact on health and longevity. Due to the fact that rs9472817 is located in intron 8, the exact molecular mechanism responsible for the effect on survival remains to be elucidated. Although it has been reported that intronic SNPs may have an effect on gene expression [94], it is more plausible for rs9472817 to be in LD with some other functional genetic alterations affecting the function of UCP4 and this has consequences on the aging nervous system, leading to the observed detrimental effect of the GG genotype. Further studies are needed to clarify this point.

In conclusion, we found that the genetic variability of *UCP* genes affects human longevity. This finding is in agreement with previous data showing that energy storage and expenditure have a key role in survival at old age and support the Uncoupling-to-survive hypothesis. Although this hypothesis was initially based on the possible effects of UCPs on the oxidative stress, the most recent data have emphasized the impact of UCP variations on the metabolic efficiency. It is then likely that the impact of *UCP*s' gene variation on longevity is due to the complexity of these functions rather than the sole effect of oxidative stress. On the other hand, due to the presence of different *UCP* genes, each active in specific tissues, further analyses will be necessary to understand the specific role of the uncoupling process in different tissues and, consequently, in correlation with differing metabolism and, possibly, nutrients.

## Supporting Information

Table S1 Genotypic and allelic frequencies in the analyzed sample by group.
(PDF)
