# Supplementary material for: Further Support to the Uncoupling-to-Survive Theory: The Genetic Variation of Human UCP Genes Is Associated with Longevity
Source: PLoS One. 2011 Dec 27;6(12):e29650. doi: 10.1371/journal.pone.0029650 (PMC3246500; doi:10.1371/journal.pone.0029650)
Supplement: Table S1 — Genotypic and allelic frequencies in the analyzed sample by group. (PDF) [file pone.0029650.s001.pdf]

Table S1. Genotypic and allelic frequencies in the analyzed sample by group

	Younger group (N=375)			Older group (N=223)		
UCP2 rs659366	Genotypes	Frequency	Percentage	Genotypes	Frequency	Percentage
	G/G	183	48.80	G/G	100	44.84
	G/A	170	45.33	G/A	107	47.98
	A/A	22	5.87	A/A	16	7.17
	Alleles	Frequency	percentage	Alleles	Frequency	Percentage
	G	536	71.47	G	307	68.83
	A	214	28.53	A	139	31.17
UCP2 rs660339	Genotypes	Frequency	Percentage	Genotypes	Frequency	Percentage
	C/C	180	48.00	C/C	83	37.22
	C/T	156	41.60	C/T	106	47.53
	T/T	39	10.40	T/T	34	15.25
	Alleles	Frequency	Percentage	Alleles	Frequency	Percentage
	C	516	68.80	C	272	60.99
	T	234	31.20	T	174	39.01
UCP3 rs15763	Genotypes	Frequency	Percentage	Genotypes	Frequency	Percentage
	C/C	218	58.13	C/C	121	54.26
	C/T	141	37.60	C/T	82	36.77
	T/T	16	4.27	T/T	20	8.97
	Alleles	Frequency	Percentage	Alleles	Frequency	Percentage
	C	577	76.93	C	324	72.65
	T	173	23.07	T	122	27.35
UCP3 rs1800849	Genotypes	Frequency	Percentage	Genotypes	Frequency	Percentage
	C/C	322	85.87	C/C	164	73.54
	C/T	48	12.80	C/T	58	26.01
	T/T	5	1.33	T/T	1	0.45
	Alleles	Frequency	Percentage	Alleles	Frequency	Percentage
	C	692	92.27	C	386	86.55
	T	58	7.73	T	60	13.45

UCP4 rs9472817	Genotypes	Frequency	Percentage	Genotypes	Frequency	Percentage
	C/C	92	24.53	C/C	58	26.01
	C/G	176	46.93	C/G	125	56.05
	G/G	107	28.53	G/G	40	17.94
	Alleles	Frequency	Percentage	Alleles	Frequency	Percentage
	C	360	48.00	C	241	54.04
	G	390	52.00	G	205	45.96
UCP4 rs10498769	Genotypes	Frequency	Percentage	Genotypes	Frequency	Percentage
	C/C	240	64.00	C/C	147	65.92
	C/G	124	33.07	C/G	71	31.84
	G/G	11	2.93	G/G	5	2.24
	Alleles	Frequency	Percentage	Alleles	Frequency	Percentage
	C	604	80.53	C	365	81.84
	G	146	19.47	G	81	18.16
UCP5 rs2235800	Genotypes*	Frequency	Percentage	Genotypes	Frequency	Percentage
	T/T	75	35.21	T/T	33	35.87
	T/A	112	52.58	T/A	46	50.00
	A/A	26	12.21	A/A	13	14.13
	Alleles	Frequency	Percentage	Alleles	Frequency	Percentage
	T	262	61.50	T	112	60.87
	A	164	38.50	A	72	39.13
UCP5 rs5975178	Genotypes*	Frequency	Percentage	Genotypes	Frequency	Percentage
	T/T	56	26.29	T/T	29	31.52
	C/T	106	49.77	C/T	45	48.91
	C/C	51	23.94	C/C	18	19.57
	Alleles	Frequency	Percentage	Alleles	Frequency	Percentage
	C	208	48.82629	C	81	44.02
	T	218	51.17371	T	103	55.98

*Genotypic and allelic data refer to only the female sample.
